# A remedy for unusual *SHELXL* weighting schemes

**DOI:** 10.1107/S2056989025007327

**Published:** 2025-09-05

**Authors:** Sean Parkin

**Affiliations:** a Department of Chemistry, University of Kentucky, Lexington KY, 40506-0055 USA; Harvard University, USA

**Keywords:** *SHELXL* WGHT, high *b*-value in WGHT, *SADABS* defaults, *I*/s(*I*) problems, data reduction

## Abstract

In routine small-molecule structure refinements with *SHELXL*, the default weighting scheme relies on two adjustable parameters, *a* and *b*, in the WGHT command. For most refinements the *a* value is typically <0.1 while *b* is expected to be <1 or so. High values of *a* can be a consequence of poor counting statistics, *i.e.*, weak data. Large values of *b* may result from uncorrected systematic errors and/or unresolved problems, such as missed symmetry, disorder, and twinning. Nevertheless, on occasion large *b* values persist, even when there are no apparent model deficiencies. In such cases, the available evidence suggests that the uncertainties derived during data reduction are too small. This short tutorial-style paper presents a straightforward means of achieving WGHT parameters within the expected ranges by fine-tuning parameters used in the Bruker data scaling, merging, and absorption correction program *SADABS*. Thus, it could also provide a basis for further exploration of weighting schemes in *SHELXL.* The general principles ought to be adaptable to data reduction software from other diffractometer manufacturers.

## Abstract 2

A remedy for unusual weighting schemes sometimes encountered in structure refinements with *SHELXL* is presented, along with a worked example.

## Introduction

A Google search for something like ‘shelxl wght’ or ‘shelxl wght problems’ returns many links that mention the WGHT command in *SHELXL* (Sheldrick, 2015 ▸). Most of these, however, merely repeat the contents of the *SHELXL* manual. Few, if any, give specific information on the cause of unusual WGHT parameters, nor any useful advice on how to deal with them. The aim of this short paper is to present one straightforward way to deal with the problem of high *b* in the optimized weighting scheme. It does not, however, claim to identify or treat the root cause. Before describing the process in detail, a brief introduction to the WGHT command and its various options is warranted. None of this first part is new of course, it is just another re-wording of the *SHELXL* manual.

The *SHELXL* weighting scheme takes the following form:

w = q/[σ^2^(*F_o_*^2^) + (a*P)^2^ + b*P + d + e*sin(θ)/λ]

Weights are applied via the WGHT command, which has six adjustable parameters:

WGHT a[0.1] b[0] c[0] d[0] e[0] f[.33333]

Here, the values given in square brackets are defaults. The value of *P* is set according to:

P = [f * maximum of (0 or F_o_^2^) + (1-f) * Fc^2^]and is intended to reduce bias (Wilson, 1976 ▸). From this assignment, the *a* parameter is quadratic in *P*, whereas *b* is linear. As a consequence, having *a* > 0 affects the weights of strong intensities to a greater degree than weak intensities, whereas *b* primarily affects the weights of weak intensities. Nonetheless, whether the use of *P* in this way is a realistic proposition has recently come into question (Henn, 2025 ▸). The value of *q* is 1 if *c* = 0, but if *c* is set to a non-zero value, the effect is to upweight the higher angle data, which can be useful during refinement for finding hydrogen atoms in difference maps *etc*. The mode of action is different for positive versus negative *c*. The *SHELXL* manual goes into more detail for specialized weighting schemes, but for most structures, WGHT requires just *a* and *b* (and sometimes only *a*). For a high-quality structure with reliable diffraction data, the expectation is for *a* < 0.1 and *b* < 1. After each round of refinement, *SHELXL* suggests new WGHT parameters with *a* and *b* optimized to flatten the analysis of variance, *i.e.*, to force the goodness-of-fit to ∼1.0. These empirically derived *a* and *b* parameters, however, are sometimes larger than expected. Large *a* values for example, can be indicative of weak data. Large values of *b* are trickier to interpret but can be due to model deficiencies and/or to problems with the reflection standard uncertainties [‘su’ or σ(*F_o_*^2^)]. Most small-molecule crystallographers will have encountered a *checkCIF* alert concerning large *b* parameters at some point, *i.e.*, something like this:

PLAT083 Type_2 Test for extreme second weighting parameter value (SHELX). The second parameter on the SHELXL weighting line has an exceptionally large value. This may indicate either improper reflection s.u.s or an unresolved problem such as missed twinning.

Assuming that all serious model deficiencies (*e.g.*, missed symmetry, twinning, disorder, *etc*.) or data-processing issues (*e.g.*, absorption correction, scan truncation, *etc*.) have been properly dealt with, are better estimates of σ(*F_o_*^2^) possible? For Bruker data, σ(*F_o_*^2^) are calculated by *SADABS* (Krause *et al.*, 2015 ▸) using information from the integration program *SAINT* (Bruker, 2023 ▸). Nowadays, both programs are usually called from within the *APEX* gui. Other manufacturers have their own programs [*e.g.*, *CrysAlis PRO* (Rigaku, 2015 ▸) and *X-RED* (Stoe & Cie, 2002 ▸), *etc*.] with analogous procedures, so the following could probably be adapted for other architectures. Any scheme to improve σ(*F_o_*^2^) values in *SADABS* will entail changing its defaults, not all of which can be accessed from the *APEX* gui, so the command-line version of *SADABS* is required. First though, scrutiny of the σ(*F_o_*^2^) values is required; are they too large, too small, or something else?

In *SHELXL*, data are input via the HKLF instruction, typically:

HKLF 4

The full form of the HKLF command, however:

HKLF N S r11 r12 r13 r21 r22 r23 r31 r32 r33 sm m

allows some manipulation of the data as it is read in by *SHELXL*. Here, *N* sets the data format, *S* scales both *F_o_*^2^ and σ(*F_o_*^2^), *r_11_*…*r_33_* is a 3×3 transformation matrix, *sm* scales just the σ(*F_o_*^2^), and *m* is for compatibility with ‘condensed data’ (ancient and obsolete). For the purpose at hand, the important parameter is *sm*. For example, if *sm* = 0.5, then all σ(*F_o_*^2^) will be halved, whereas *sm* = 2 would double all the σ(*F_o_*^2^). Thus, a quick test should reveal whether the σ(*F_o_*^2^) are too small or too large. This is best illustrated by an example.

## Diagnosis of σ(*F_o_^2^*) problems

**Figure 1:**
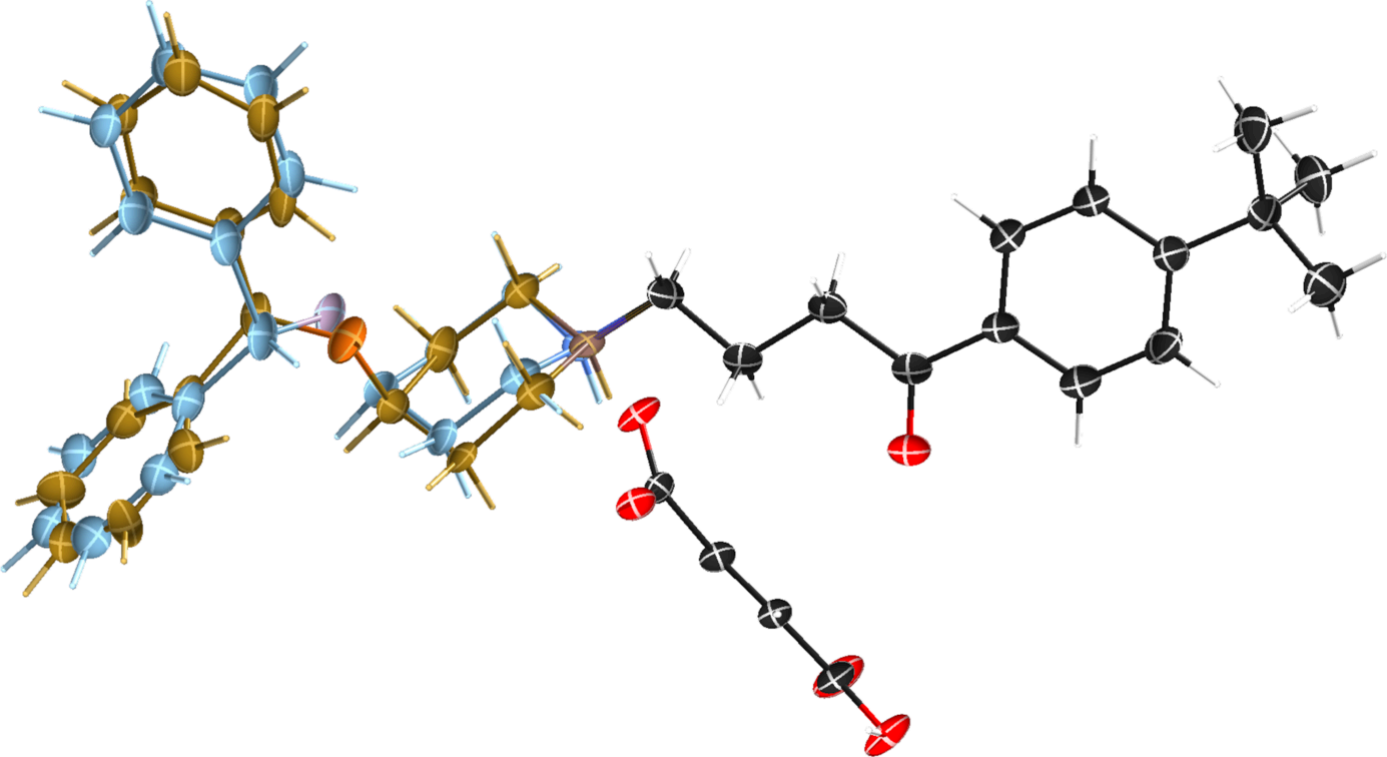
An ellipsoid plot (50% probability) of ebastinium fumarate showing the extensive disorder present in over half the main molecule (left-hand side).

The effect of the *SHELXL* HKLF parameter ‘*sm*’ on suggested WGHT parameters can be ascertained from an example structure. Any reasonable quality crystal structure is suitable for this exercise. The structure of ebastinium fumarate (Priyanka *et al.*, 2022 ▸), Fig. 1 ▸, will suffice.

There is nothing particularly special about this structure, but it did have a few common problems that needed to be dealt with prior to investigating its weighting scheme. All available crystals were twinned by pseudo-merohedry (Parkin, 2021 ▸), though careful surgery with a razor blade reduced the minor component to a tiny but not insignificant sliver. It also had extensive disorder. These issues had to be addressed before looking into the weights, as model deficiencies are known to affect the suggested WGHT parameters in *SHELXL* (see above). The *.hkl and *.res files are available in the supporting information. The final refinement of the structure had WGHT parameters optimized as follows:

WGHT 0.033300 1.537000

There’s nothing too unusual there. The space group is *P*2_1_/*c* but the data had been collected as if it were *P*2_1_/*a*, so must be transformed on input using *r_11_*…*r_33_* on the HKLF command. A refinement run with *sm* = 0.5 will show how the suggested *a* and *b* parameters are affected by overly small estimates of σ(*F_o_*^2^), *i.e.*, using

HKLF 4 1 0 0 1 0 -1 0 1 0 0 0.5

results in:

WGHT 0.0210 2.6880

Here, the *a* parameter is reduced, but *b* increases by about 75%. What if *sm* is set to 1.5?

HKLF 4 1 0 0 1 0 -1 0 1 0 0 1.5

The suggested WGHT becomes:

WGHT 0.0402 0.0000

Now *a* is a bit larger but the *b* value has been driven down to zero. Thus, increasing the σ(*F_o_*^2^) values increased *a* slightly but reduced *b* dramatically. The upshot is that, in the absence of model deficiencies or other problems, large *b* parameters in WGHT are caused by *underestimated σ*(*F_o_*^2^). It also means that the default values in *SADABS* are not always appropriate. One might assume that simply using *sm* > 1 on the HKLF command to increase all σ(*F_o_*^2^) by the same factor might fix the unusual WGHT problem, but that would affect all reflections in the same way. For a reason why that is unlikely to be optimal, see the last paragraph of Section 3, below. Either way, facilities within *SADABS* for generating σ(*F_o_*^2^) are more flexible than, and preferable to, a simple multiplier like *sm*. Thus, command-line *SADABS* is required. Prior to that, however, a brief review of the options available in *SADABS* is in order.

## Options for σ(*F_o_^2^*) in *SADABS*

In *SADABS*, intensity standard uncertainties [*i.e.*, su or σ(*F_o_*^2^)] are assigned as follows:

su^2^ = [K*σ(I)]^2^ + [g<I>]^2^where the σ(*I*) are from *SAINT* and account for counting statistics and background *etc. SADABS* provides twelve ways to specify *K* and *g*, as follows:

[0] K = 1, g = 0

[1] K = 1, refine overall g

[2] K = 1, refine all g

[3] refine overall K and overall g

[4] refine overall K and all g

[5] refine all K and overall g

[6] refine all K and all g

[7] refine overall K, input fixed g

[8] refine all K, input fixed g

[9] input fixed K, refine overall g

[10] input fixed K, refine all g

[11] input fixed K and g

Here, for the refined values, ‘all’ would refine separate values of *K* and/or *g* for each individual data-collection scan, whereas ‘overall’ would refine single values for all included scans. The default is option [5], which as previously shown, sometimes results in *SHELXL* weights with larger than expected *b* values. The *SADABS* *.abs file lists assigned (or refined) values of *K* and *g*. The *K* parameter acts as a scale factor applied to σ(*I*), so it is similar in scope to *sm* in the (expanded) HKLF command. As shown above, refinement of *K* is not optimal in all cases, as it can refine to too low a value. Options [9], [10], and [11], however, allow input of fixed *K*. For a reasonable first guess at *K*, one could try the *sm* value obtained on an HKLF test, as conducted in Section 2, above. What then for parameter ‘*g*’? The *g* value is simply the reciprocal of the limiting value of *I*/σ(*I*) for a reflection of infinite intensity. In visual terms, it sets the upper limit of the Diederichs plot (Diederichs, 2010 ▸). Thus, a default *SADABS* run is intended to give a value for *g* that is optimal for the dataset at hand. At the very least, it’s the best (*i.e.*, only!) estimate available. Moreover, the most intense reflections are more likely to have reasonable *I*/σ(*I*) values; it’s the weaker reflections that are most severely affected, leading to large *b*-values in *SHELXL* weights. Attempts to refine *g* (options [9] or [10]) would inevitably alter the limiting *I*/σ(*I*), which without good reason is not justified [note: this is also the reason why simply adjusting the *sm* parameter on the HKLF line to re-scale the σ(*F_o_*^2^) values is not recommended]. This all points to option [11] as the appropriate choice, as illustrated below.

## A *SADABS* remedy for large *b* in *SHELXL* WGHT

**Figure 2:**
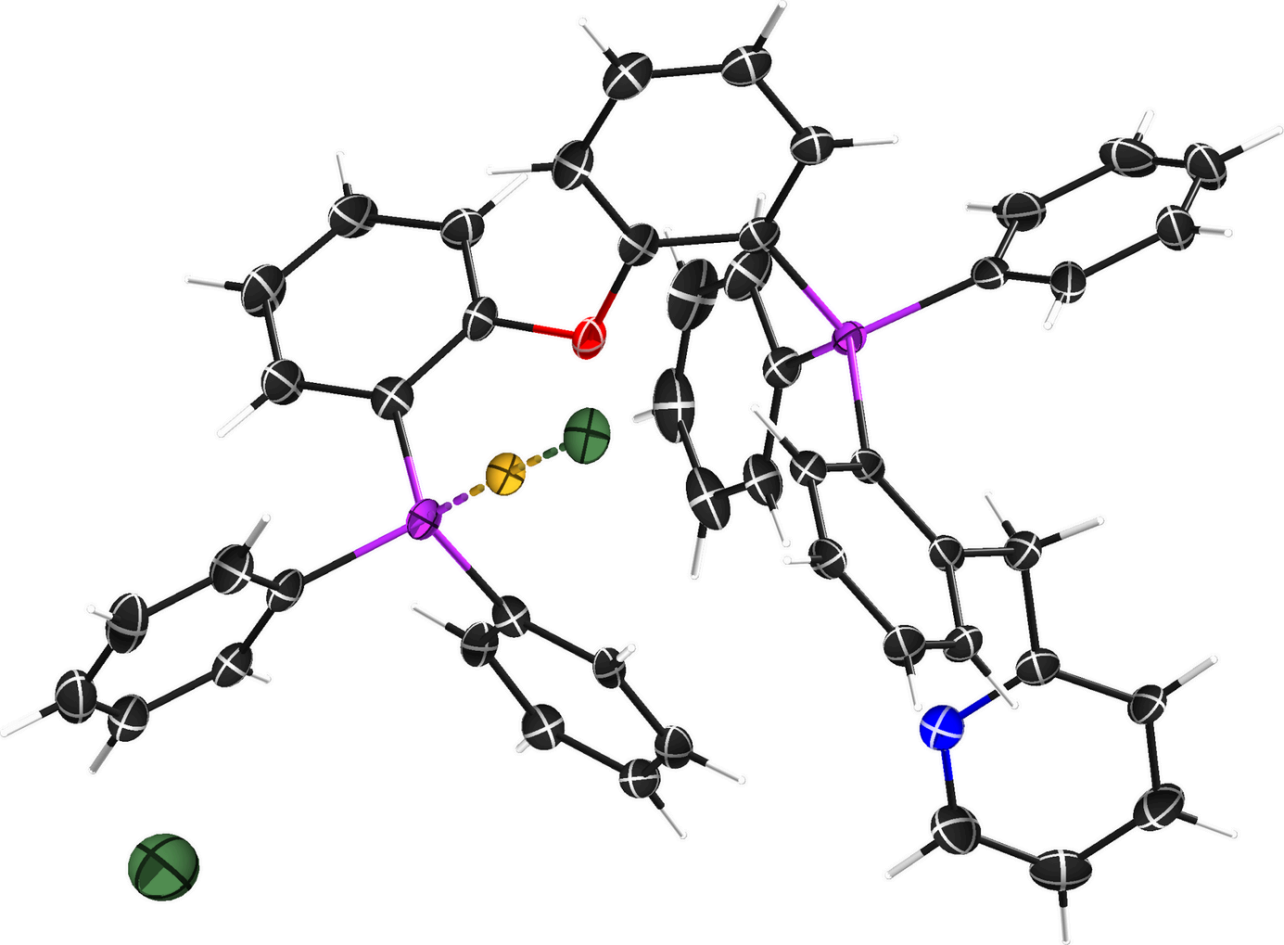
An ellipsoid plot (50% probability) of gold complex STG-2.

**Figure 3:**
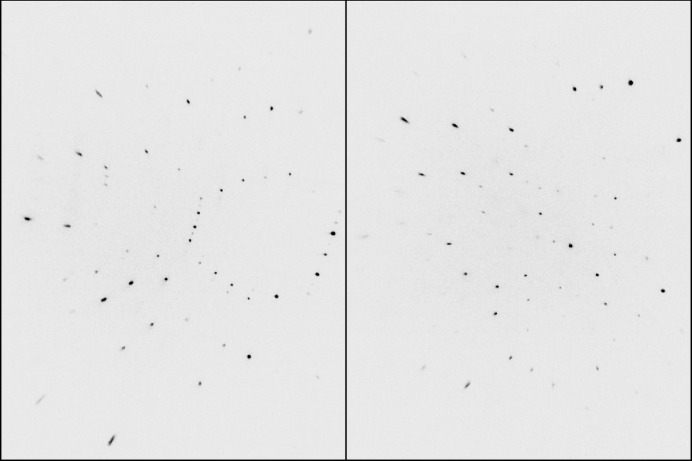
Representative data frames from gold complex STG-2 in two different regions of reciprocal space. Nothing seems to be amiss.

A recently published gold-containing complex, ‘STG-2’ (Gilpatrick *et al.*, 2024 ▸) (Fig. 2 ▸) that gave particularly unusual WGHT parameters will be used. This was a Cu *Kα* dataset, which is not ideal for a gold compound (the Mo *Kα* optics were out for repair at the time), but diffraction images all looked quite reasonable (see Fig. 3 ▸) and gave no obvious indication of problems. The published STG-2 made use of *SQUEEZE* (Spek, 2015 ▸) but was otherwise well behaved, so the unusual WGHT parameters were a surprise; related structures were not similarly affected. The *.raw, *.hkl, *.fab, and *.res files for STG-2 are available in the SI.

Use of default values forced by the *APEX* gui version of *SADABS* gave *K* in the range 0.817–0.984 and a refined *g* of 0.0447, which in turn gave the following WGHT parameters for a fully refined model:

WGHT 0.000000 83.604500

**Figure 4:**
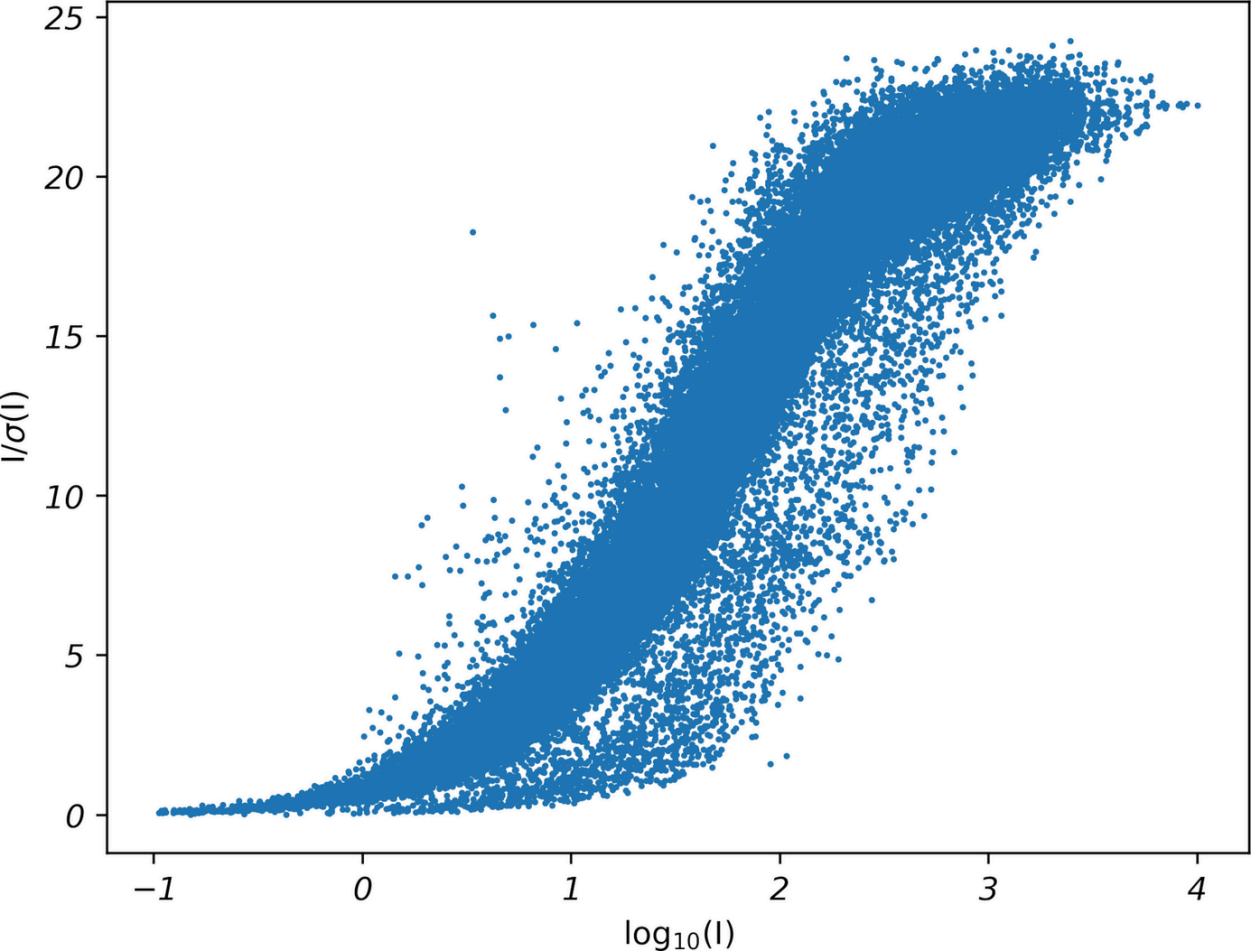
A Diederichs plot for STG-2 from a default *SADABS* run. Note the abundance of points at the upper right [high *I*/σ(*I*)] of the scatterplot. The sparsely populated region to the right of the main sequence is due to inclusion of ‘fast scan’ reflections, used to replace overloads in the main scans.

This default *SADABS* run gave the Diederichs plot shown in Fig. 4 ▸, indicating a limiting *I*/σ(*I*) of 22.4. For context, values quoted by Krause *et al.* (2015 ▸) range between 16.4 and 82.5, and the authors suggest a value of ∼30 being ‘good for synchrotron data’. Thus, the overall counting statistics for STG-2 seem reasonable for a routine structure.

After a bit of experimentation, setting *sm* = 3.4 as the 12th parameter on the HKLF command subsequently led to:

WGHT 0.0000 0.0620

The *a* parameter remains at zero and *b* is now within the expected range. The question is whether something similar can be achieved using command-line *SADABS*.

Given the above *sm* trials, an initial test with fixed *K* = 3 and *g* = 0.0447 is reasonable. It invariably takes some iteration to optimize, so it helps to give each *SADABS* trial a separate filename (*e.g.*, ‘fix-K3-g’). For this first trial, salient parts of the screen output from *SADABS* are reproduced below with brief explanations where necessary. The input raw data filename "c24009" is just the sequence number assigned to STG-2 when the data were collected. The next few text snippets show the screen output of the command-line *SADABS* run (highlights indicate changes from defaults).

SADABS-2014/4 - Bruker AXS area detector scaling and absorption correction

---------------------------------------

Note that all questions except those asking for a filename *etc*. may be answered by "Q" to force SADABS to terminate immediately.

Expert mode (Y or N) [N]: Y

Maximum number of reflections allowed (2000000):

Enter listing filename [sad.abs]: fix-K3-g.abs

--- lines omitted ---

Enter Laue group number [2]:

Choose ‘expert mode’ to access to all facilities within *SADABS* and change the name of the log (.abs) file. The next few queries by *SADABS* can (for this structure) use defaults, until:

Treat Friedel opposites as equivalent for parameter refinement (Y or N)?

Answering "N" is not recommended unless you have a high redundancy [Y]:

--- lines omitted ---

Enter filename (/ if no more) []: c24009.raw

Enter filename (/ if no more) [c24010.raw]:

** Cannot open file c24010.raw

** Enter filename (/ if no more) []:

Here, enter the raw data filename created by *SAINT* during integration. The next few *SADABS* inputs may usually be given default values, so are not reproduced here. Strongly absorbing crystals, however, might require custom entries. The next required changes are to specify *K* and *g*:

K = 1, g = 0 (0), K = 1, refine overall g (1), K = 1, refine all g (2), refine overall K and overall g (3), refine overall K and all g (4), refine all K and overall g (5), refine all K and all g (6), refine overall K, input fixed g (7), refine all K, input fixed g (8), input fixed K, refine overall g (9), input fixed K, refine all g (10), input fixed K and g (11) [5]: 11

Enter value for K [1]: 3

Enter value for g [0.03]: 0.0447

Choose option [11], fix *K* = 3, and *g* = 0.0447, which results in the following:

Run 2th R(int) Incid. factors Diffr. factors K g I/s(lim) Total I>2sig(I) 1 55.0 0.0429 0.842 – 0.922 0.894 – 1.114 3.000 0.0447 22.4 4437 2885 2 107.7 0.0361 0.873 – 0.994 0.897 – 1.273 3.000 0.0447 22.4 5898 4044

--- lines omitted ---

12 − 48.1 0.0332 0.849 – 1.051 0.895 – 1.359 3.000 0.0447 22.4 3928 3099 13 107.7 0.0391 0.852 – 0.973 0.897 – 1.329 3.000 0.0447 22.4 4928 3587

Notice that the *I*/σ(lim) value is just the reciprocal of *g*, *i.e.*, 22.4 = 1/0.0447. The next few queries can usually be met with defaults, but the custom filename helps with bookkeeping:

PART 3 - Output Postscript diagnostics and corrected data

--- lines omitted ---

Reflection output file [sad.*hkl*]: fix-K3-g.hkl

Mu*r of equivalent sphere for additional absorption correction [0.2]: 0.41

Aside: Here, the *μR* of an ‘equivalent sphere’ for approximating the 2*θ*-dependent component of the absorption surface is also changed. The recommendation given by Krause *et al.* (2015 ▸) is to bias the ‘equivalent sphere radius’ towards the smallest crystal dimension, but they give no explicit information on how strong such a bias should be. They do, however, provide an example in which a crystal of size 0.3 × 0.2 × 0.1 mm with μ = 10 mm^−1^ is assigned an ‘effective sphere radius’ of 0.07 mm. Taken at face value, their example upweights the smallest crystal dimension by ∼5.5 times, *viz*.

(0.1x + 0.2 + 0.3)/[2(x + 2)] = 0.07

0.1x + 0.5 = 0.07(2x + 4)

0.1x - 0.14x = 0.28–0.5

x = (0.1–0.14) / (0.28–0.5)

x = 5.5

Here, the value of x is the factor by which the smallest crystal dimension is upweighted. Thus, the numerator in the first equation corresponds to the weighted sum of crystal dimensions, while the denominator is the sum of the weights. This approximation works surprisingly well in most cases, is easy to calculate, and may also be used in the *APEX* gui implementation of *SADABS*. At the very least, it gives an excellent starting estimate for further (manual) optimization. For STG-2 the crystal used was 0.20 × 0.15 × 0.07 mm and had μ = 8.42 mm^−1^, therefore:

*μR* = [(5.5 * 0.07) + 0.15 + 0.20] * 8.42) / 2(5.5 + 2) = 0.41

giving *μR* = 0.41 for an ‘effective sphere radius’ of 0.049 mm. As a result, one might consider experimentation with higher-order spherical harmonics for the absorption correction.

The result is a newly written dataset ‘fix-K3-g.hkl’ and its attendant log (*.abs) file. The previously refined model may then be used (for STG-2 the *.fab file is also needed since it used *SQUEEZE*) to iterate the refinement until the WGHT parameters converge. This can be done manually, but is easily automated using *ShelXle* (Hübschle *et al.*, 2011 ▸) and presumably other programs. Such a test gave the following:

WGHT 0.0300 44.0202

The resulting weights appear to be heading in the right direction. The *a* parameter is <0.1 and *b* is roughly halved but is still too large; subsequent trials will increase *K*, *e.g.*, starting at *K* = 4. There’s no need to explicitly present all the *SADABS* output again: simply repeat the above steps but use *K* = 4, *g* = 0.0447, and assign a filename something like ‘fix-K4-g’, which gives:

WGHT 0.0348 11.6088

**Figure 5:**
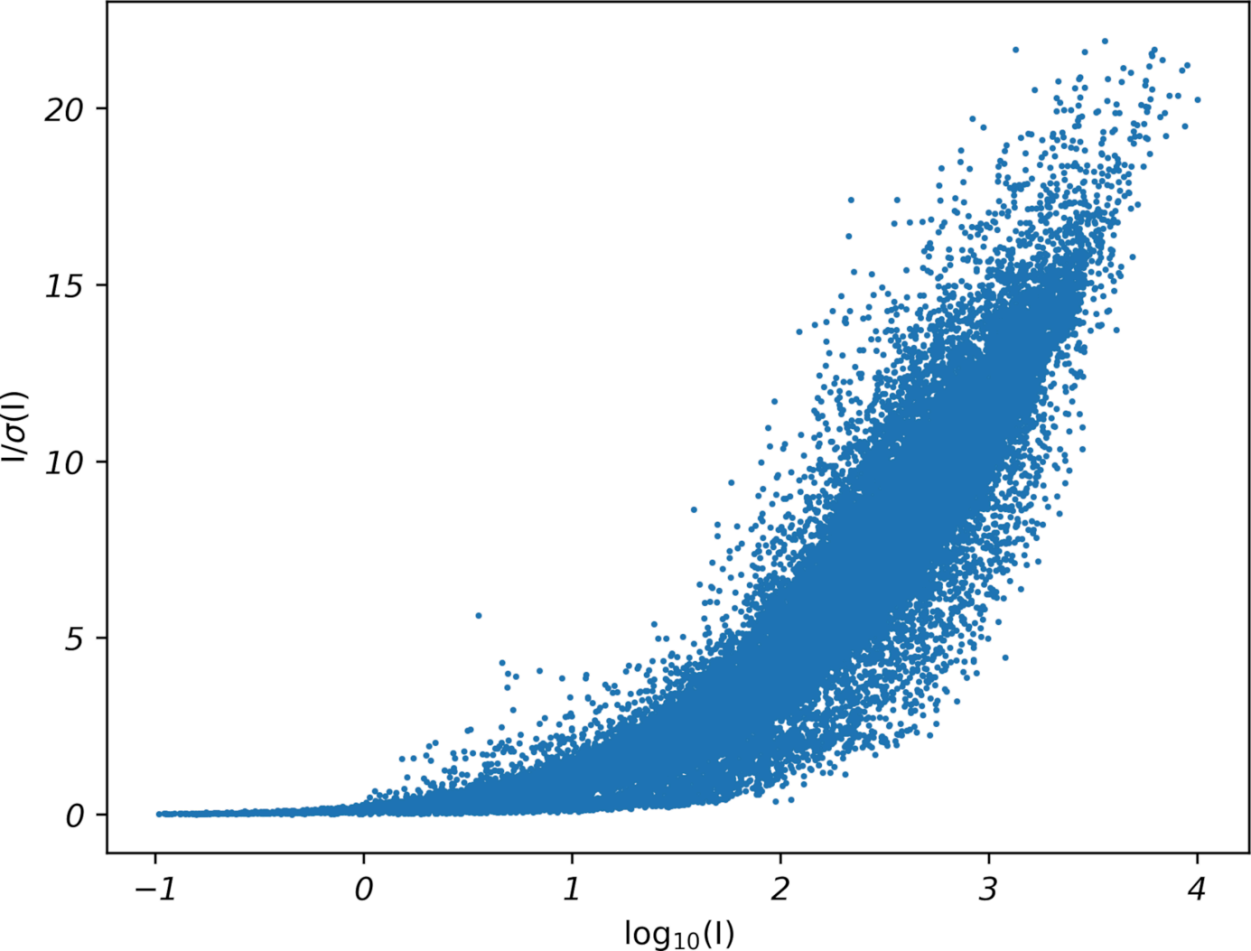
A Diederichs plot for STG-2 from a *SADABS* run using *K* = 4.3 and *g* = 0.0447.  The number of points at high *I*/σ(*I*) is much reduced compared to a default *SADABS* run (Fig. 4 ▸), as expected.

This is still going in the right direction. After running a few more trials with *K* increasing by 0.1 each time (see Table 1 ▸), it looks as though *K* of about 4.3 in *SADABS* produces WGHT parameters within expected ranges and any further increases in *K* are overkill. This is also approximately the point where the *a* parameter is maximized, though whether this is a significant (or even general) observation requires further investigation. A Diederichs plot for the dataset resulting from *K* = 4.3, *g* = 0.0447 is shown in Fig. 5 ▸. Note that the height of the Diederichs plot in Fig. 5 ▸ matches that of the default run in Fig. 4 ▸, but many of the points are shifted to lower *I*/σ(*I*), leaving the plot a little sparse in the upper right. The treatment of fast scans within *SAINT* and *SADABS*, however, is unclear. Nonetheless, the general shift to lower *I*/σ(*I*) is expected, as the σ(*F_o_*^2^) had been previously diagnosed as *underestimated*. The WGHT *b* parameter suggested by *SHELXL* for STG-2 is thus now within the expected range, while preserving most other details, including the ‘flatness’ of the analysis of variance (see *.lst file) and the overall *I*/σ(limit). Subsequent refinement using this dataset in *SHELXL* led to suggested WGHT parameters of *a* = 0.0364, *b* = 0.000.

**Figure 6:**
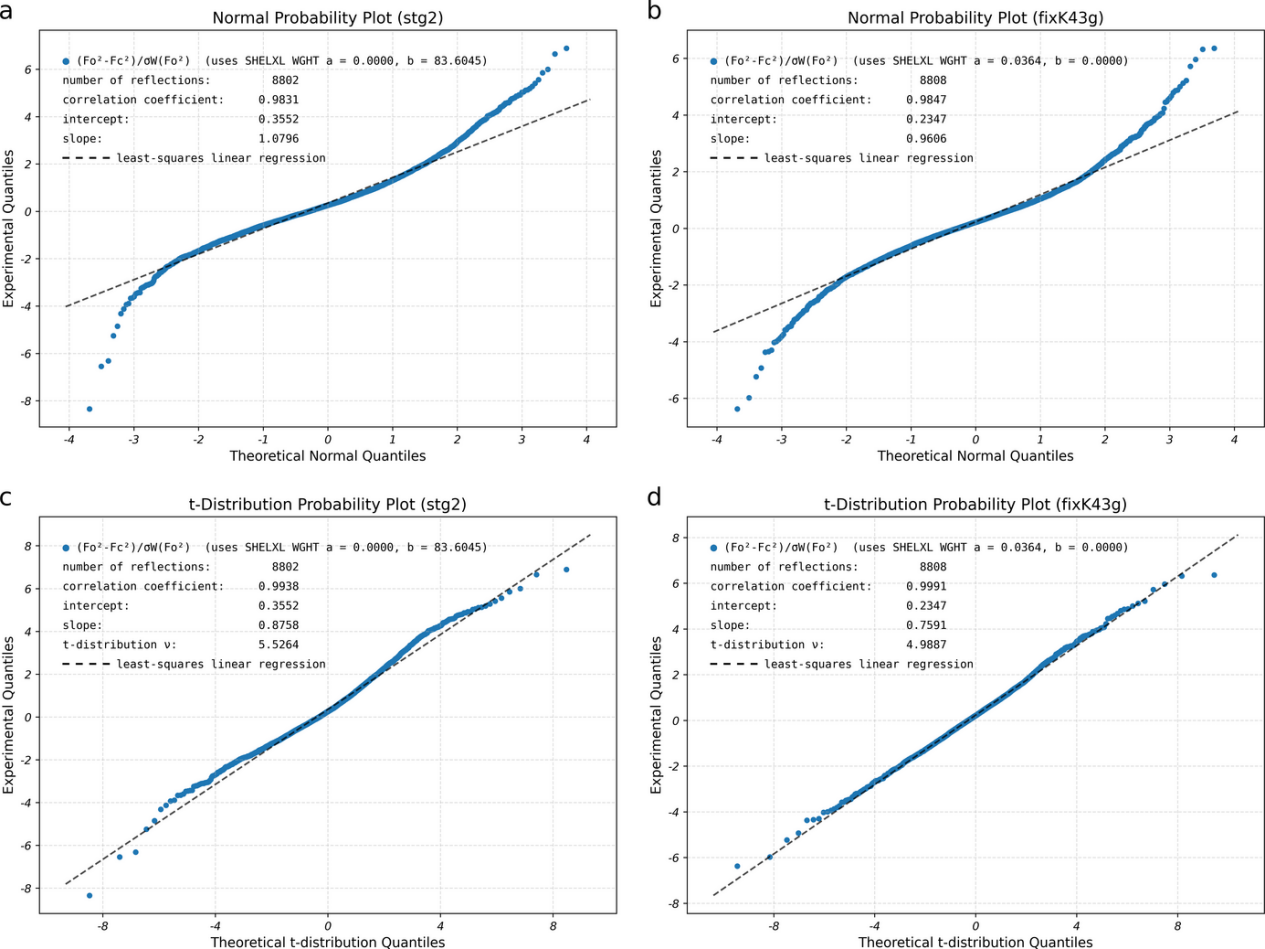
Probability plots for STG-2: (*a*) normal probability plot (npp) using default *SADABS* data, (*b*) npp using manually optimized *SADABS* data, (*c*) Student’s t-distribution probability plot (tpp) for default *SADABS* data, (*d*) tpp with manually optimized *SADABS* data. Note that for either type of distribution, the non-default plots (panels *b* and *d*) are more symmetric.

**Figure 7:**
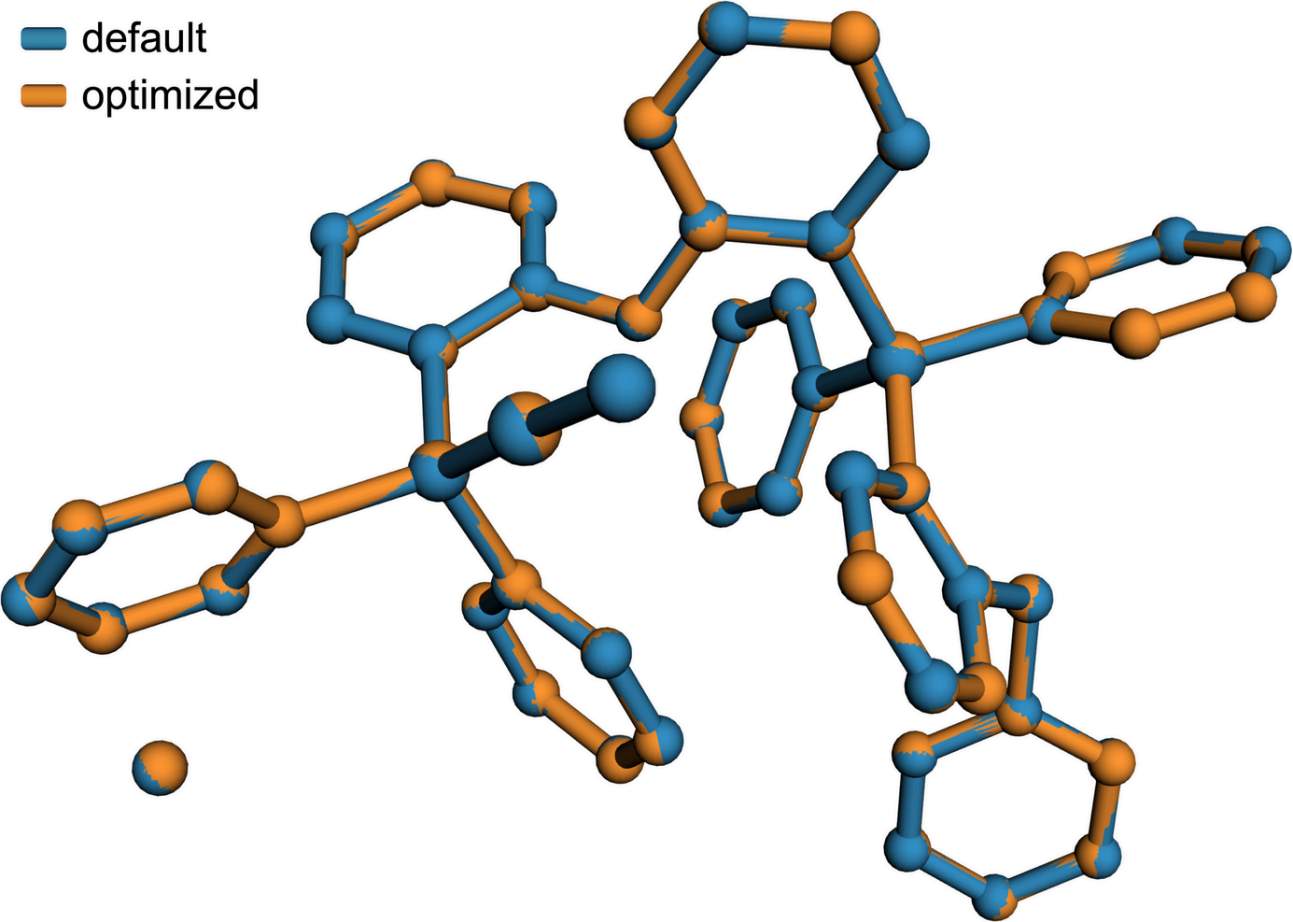
An overlay of models for STG-2 refined using default (blue) and optimized (orange) *SADABS*-derived data. The RMSD for all corresponding atom pairs is negligible at 0.0045 Å.

One remaining question is whether these changes represent actual improvements or if they are merely cosmetic. Some statistics for STG-2 obtained from refinement using the original (default *SADABS* parameters) and optimized (as per this work) are given in Table 2 ▸. The changes are modest, but better across the board for the non-default *SADABS*-generated dataset. Further evidence may be provided by normal probability plots (npp) advocated by Abrahams & Keve (1971 ▸) obtained from the results of these refinements. In such npp [Fig. 6 ▸(*a*),(*b*)], the ideal correlation coefficient, slope, and intercept would be 1.0, 1.0, and 0.0. In each case, the optimized *SADABS* parameters lead to modest improvements, which are also summarized in Table 2 ▸. The curvature observed in the npp at the high and low extremes result from the uncertainties not being normally distributed. This point is rarely discussed, but as pointed out by Hooft *et al.* (2009 ▸), it is a common occurrence in crystallographic results, and suggests that probability plots based on Student’s t-distribution (tpp) would better model the uncertainties. In these tpp [Fig. 6 ▸(*c*),(*d*)], the fit is indeed considerably more linear, but the slopes are less than 1.0, a point also noted by Hooft *et al.* (2009 ▸) in their own examples. Python code used to prepare all the probability plots in Fig. 6 ▸ is available in the supporting information (program tnpp.py). It is worth noting that little of this makes much difference to the resulting atomic coordinates, as is evident from an overlay plot of the two models (Fig. 7 ▸; Parkin, 2025 ▸), for which the RMSD over all atom pairs is only 0.0045 Å. Indeed, the most immediate benefit is removal of an otherwise intractable *checkCIF* alert. This (STG-2) was an extreme case: few large-*b* WGHT problems require such a high *K* value in *SADABS*. Something like *K* = 1.1–1.3 is more typical.

## Final words

It is important to state that the root cause of underestimated σ(*F_o_*^2^) is not addressed here. Most likely that is buried deep in some proprietary routine in the integration program. Back in the days of serial diffractometry, algorithms designed to minimize σ(*I*)/*I* (and thereby maximize signal:noise) could cause scan truncation, leading to underestimated intensities (and presumably their su) (Lehmann & Larsen, 1974 ▸). Nowadays, from a manufacturer’s perspective, claims of ‘lowest detector noise’ or even ‘zero detector noise’ make more compelling marketing jargon than ‘gives proper estimates of σ(*F_o_*^2^)’. Nevertheless, the method presented here provides a straightforward and logical means of dealing with unusual *SHELXL* weighting schemes, simply by changing a few default parameters.

## Supplementary Material

Files necessary to reproduce the trials involving ebastinium fumarate and gold complex STG-2, plus a python program for making Diederichs plots are included in the SI. This file is ebastinium.hkl. DOI: 10.1107/S2056989025007327/oi2020sup2.txt

ebastinium.res. DOI: 10.1107/S2056989025007327/oi2020sup3.txt

stg2.raw. DOI: 10.1107/S2056989025007327/oi2020sup4.txt

stg2.hkl. DOI: 10.1107/S2056989025007327/oi2020sup5.txt

stg2.fab. DOI: 10.1107/S2056989025007327/oi2020sup6.txt

stg2.res. DOI: 10.1107/S2056989025007327/oi2020sup7.txt

Archive containing python code ddrch.py. DOI: 10.1107/S2056989025007327/oi2020sup8.zip

Python code for normal and t-distribution probability plots. DOI: 10.1107/S2056989025007327/oi2020sup9.zip

## Figures and Tables

**Table 1:** *SHELXL* WGHT *a* and *b* parameters resulting from *SADABS K* values for STG-2

*K*	*a*	*b*
refined	0.0000	83.6045
3.0	0.0300	44.0202
4.0	0.0348	11.6088
4.1	0.0354	7.9550
4.2	0.0358	4.4587
4.3	0.0363	0.9453
4.4	0.0355	0.0000
4.5	0.0343	0.0000

**Table 2:** Some refinement statistics for STG-2 using default and optimized *SADABS* runs

	*default K*, *g*	*optimized K*, *g*
WGHT *a, b*	0.0000, 83.6045	0.0364, 0.0000
*goof*	1.191	1.045
*R*_1_ [*I* > 2σ(*I*)]	0.0386	0.0351
*wR*_2_ (all)	0.0878	0.0875
Δρ_max_ (e Å^−3^)	0.870	0.803
Δρ_min_ (e Å^−3^)	−1.237	−1.081
C—C precision (Å)	0.0065	0.0061
*npp^a^* CC*^b^*	0.9831	0.9847
*npp^a^* slope	1.0796	0.9606
*npp^a^* intercept	0.3552	0.2347

Notes: (*a*) npp = normal probability plot (see Fig. 6 ▸); *b* CC = correlation coefficient.
